# Protocol for a community-based, household-randomised, dose–response trial to assess the acceptability, nutritional effects and safety of double-fortified salt containing iodine and folic acid compared with iodised salt among non-pregnant Ethiopian women of reproductive age (DFS-IoFA)

**DOI:** 10.1136/bmjopen-2024-084494

**Published:** 2024-10-29

**Authors:** Kenneth H Brown, Masresha Tessema, Christine M McDonald, Isaac Agbemafle, Meseret Woldeyohannes, Mengistu Fereja, Debritu Nane, Charles D Arnold, Feyissa Challa Waka, Biniyam Tesfaye, Mandana Arabi, Homero Martinez

**Affiliations:** 1 Department of Nutrition and Institute for Global Nutrition, University of California Davis, Davis, California, USA; 2 Food Science and Nutrition Research Directorate, Ethiopian Public Health Institute, Addis Ababa, Ethiopia; 3 Department of Pediatrics, University of California San Francisco, San Francisco, California, USA; 4 Department of Nutrition, University of Rhode Island, Kingston, Rhode Island, USA; 5 University of Health and Allied Sciences, Hohoe, Ghana; 6 Global Technical Services, Nutrition International, Ottawa, Ontario, Canada

**Keywords:** Epidemiology, Nutrition & Dietetics, Preventive Medicine, Public Health

## Abstract

**ABSTRACT:**

**Introduction:**

The prevalence of neural tube defects (NTDs) is higher in Ethiopia than most other countries, and ~84% of Ethiopian women of reproductive age (WRA) have folate insufficiency, a major risk factor for NTDs. Salt fortification with folic acid is a potential strategy to improve women’s folate status, but data are needed on the acceptability, nutritional impact and safety of folic acid fortification of iodised salt.

**Methods and analysis:**

The study is designed as a community-based, household-randomised, dose–response trial. A total of 360 non-pregnant WRA 18–49 years of age will be randomly assigned to one of three intervention arms: (1) iodised salt fortified with 30 ppm folic acid to provide ~200 µg folic acid/day; (2) iodised salt fortified with 90 ppm folic acid to provide ~600 µg folic acid/day; or (3) iodised salt (comparator). The preweighed salts will be delivered to participants’ homes biweekly for 26 weeks; unused salt will be collected and weighed. Fasting, venous blood samples will be collected at baseline, end line and a randomly assigned intermediate time point for assessment of folate, iodine, vitamin B_12_ and other micronutrient status biomarkers. Women’s dietary intakes, including discretionary salt consumption, will be measured using weighed food records; 24-hour urine specimens will be analysed for sodium and iodine excretion. Primary outcomes are women’s consumption of study salts, change in biomarkers of folate and iodine status and prevalence of adverse events. Results will be analysed using analysis of covariance models to estimate group mean differences for continuous outcomes, controlling for baseline measurements, and log-binomial or modified Poisson regressions for categorical outcomes. Prespecified effect modifications will be explored.

**Ethics and dissemination:**

The study has been approved by the Ethiopian Public Health Institute’s Institutional Review Board, and the protocol has been registered with ClinicalTrials.gov (registration number NCT06223854). Study results will be published in open access scientific journals and disseminated nationally in Ethiopia.

**Trial registration number:**

NCT06223854.

**Strengths and limitations of this study**

- Randomised intervention design.
- Adequate sample size to detect important differences by study arm.
- Multiple methods for assessing adherence, impact on folate and iodine status and possible adverse effects.
- Exploratory analyses of possible modifying effects of initial folate and vitamin B_12_ status, genetic polymorphisms, household food insecurity and other baseline characteristics.
- Inability to assess the effects of the intervention on household members other than women of reproductive age; and exclusion of women who are pregnant or have selected illnesses, which undermines possible extrapolation of study results to these population subgroups.

## Introduction

 The prevalence of neural tube defects (NTDs) in Ethiopia is very high relative to other countries, as described in more detail below. One of the most widely recognised and important causes of these devastating abnormalities is maternal folate insufficiency during the early weeks of pregnancy. This research project is designed to assess the effects of fortifying iodised salt (IS) with folic acid to improve women’s folate status and thereby reduce the risk of NTDs. Folic acid is a synthetic, relatively stable, oxidised form of folate (vitamin B_9_, pteroylmonoglutamate), which can be converted to reduced folate in the body. The present manuscript summarises information on the planned intervention trial, as described in detail in the master protocol (version 7, dated 16 January 2024), which covers both the previously completed, preliminary studies and the currently presented intervention trial.

NTDs, including anencephaly and spina bifida, are malformations of the brain and spinal cord which can result in spontaneous abortions, elective termination of pregnancy, stillbirths and long-term disabilities, including hydrocephalus and related central nervous system infections.^1^ In lower resource settings, approximately three-fourths of NTD-affected newborns die before the age of 5 years.^2^ Recent analyses indicate that an estimated 260 100 pregnancies worldwide were affected by NTDs in 2015, equivalent to a prevalence of 18.6 per 10 000 live births.^2^ The prevalence of NTDs is strikingly higher in Ethiopia than the global average. Two recent meta-analyses of hospital-based studies found a mean birth prevalence of 63.3 per 10 000 (95% CI 50.9, 75.7) and 59.7 per 10 000 (95% CI 42.1, 84.7),^3 4^ and one hospital-based study in Addis Ababa, which also included pregnancy terminations, found a prevalence of 127.9 per 10 000 pregnancies after 12 weeks of gestation.^5^ One study of the causes of fetal and young child deaths in eastern Ethiopia found that NTDs were the second leading cause of stillbirths, accounting for 17% of the 114 stillbirths examined.^6^

NTDs can be induced by environmental exposures, such as undernutrition and xenobiotics, genetics or a combination of these causes. There is considerable evidence from observational studies and intervention trials that low maternal folate status during the periconceptional period is a primary risk factor for NTDs.^7^,^9^ A meta-analysis of the effects of periconceptional oral folate supplementation (≥400 µg/day) that included four trials and 6708 births found that daily folic acid supplementation (alone or in combination with other vitamins and minerals) reduced the incidence of NTDs by 69% compared with no intervention, placebo or vitamins and minerals without folic acid.^10^ WHO guidelines state that red blood cell (RBC) folate concentrations can be used as an indicator of NTD risk at the population level, and WHO recommends that the RBC folate concentration of all women of reproductive age (WRA) should be greater than 906 nmol/L to minimise the risk of folate-related NTDs.^11^ This cut-off is equivalent to a cut-off of 748 nmol/L using the updated microbiological assay supported by the US Centers for Disease Control and Prevention (CDC).^12^ In Ethiopia, 84% of WRA have an RBC folate concentration less than this cut-off.^13^

In 1992, the US Public Health Service stated that all women who are capable of becoming pregnant should consume 400 µg folic acid per day to reduce their risk of having an NTD-affected pregnancy,^14^ and the US Preventive Services Task Force subsequently recommended supplementation with 400–800 µg folic acid per day.^15^ However, because maternal folate status must be sufficient before conception to prevent NTDs, as well as the facts that many pregnancies are unplanned and adherence to daily supplementation over decades is very challenging, food fortification has been proposed as a better option to deliver additional folic acid. In 1998, the US Food and Drug Administration (FDA) began mandating folic acid fortification of enriched grain products (140 µg folic acid/100 g), with a primary focus on wheat flour fortification.^16^ In 2009, WHO recommended fortification of cereal flour with folic acid to prevent NTDs, with proposed fortification levels ranging from 100 µg to 500 µg of folic acid per 100 g flour (1–5 ppm), depending on the amount of usual flour consumption by the target population.^17^ A total of 69 countries now have fortification standards for the inclusion of folic acid in wheat and/or maize flour.^18^ Notably, studies in multiple countries have consistently shown that the prevalence of NTDs declined following the implementation of folic acid fortification programmes.^19^

Flour fortification is not a viable option to achieve high folic acid coverage in Ethiopia, as only 39% of the wheat flour is industrially produced^18^; and, according to preliminary results from the latest national food consumption survey, only 30% of women have access to fortifiable cereal flour.^20^ Thus, other potential fortification vehicles need to be considered. The Ethiopian government has mandated salt iodisation since 2011, and almost all households now have access to IS.^20^ The Ethiopian FDA has authorized 19 salt iodisation plants, with a total production capacity of 228 000 tons of salt per month.^21^ The iodisation process employs spray technology to apply potassium iodate solution to achieve a target iodine concentration of 30–40 ppm in salt. Research indicates that it is technically feasible to combine folic acid in the same spray solution used for salt iodisation, and the fortificants are stable in the finished salt for at least 12 months.^22^ Preliminary studies have found that double-fortified salt (DFS) containing iodine and folic acid (IoFA) is generally acceptable in both rural and urban Ethiopian communities (B Tesfaye, unpublished). Thus, it should be possible to build on the existing salt iodisation programme to deliver folic acid.

Recognising the alarmingly high prevalence of NTDs and the high prevalence of folate insufficiency, in 2019 the Ethiopian government published a set of recommendations on specific approaches to improve women’s folate status.^23^ Among the proposed activities, the government indicated that salt fortification may be considered as an alternative to wheat flour fortification; but information is needed on the acceptability, effectiveness and safety of salt fortification with folic acid. The current study is designed to fill this information gap. The main objectives of the study are to assess among non-pregnant Ethiopian WRA: (1) the acceptability and intake of DFS containing IoFA, compared with IS; (2) the effects of DFS on biomarkers of folate and iodine status; and (3) the occurrence of any serious adverse events in relation to salt type.

## Methods and analysis

### Study design, study site and randomisation

The study is designed as a prospective, community-based, triple-blind, dose–response trial. Non-pregnant women and their households will be randomly assigned at the household level to one of three intervention arms using the Moses-Oakford block randomisation algorithm.^24^ Participant identification numbers will be assigned sequentially by the field team, as described below.

The study team will monitor the women’s salt consumption, but will not control their intakes, so the study is viewed as a ‘hybrid’ efficacy/effectiveness trial. The trial is designated as such because the protocol does not employ a true efficacy design (in which salt intakes would be controlled), nor does it conform to a true effectiveness design (in which the participants would acquire the salt independently under usual market conditions).

The study salts will be coded by participant identification number, and the study participants, data collectors, laboratory and data analysts, and investigators will not be informed of the group identity until data collection and preliminary analyses are completed. However, because of subtle changes in the colour of the folic acid-fortified salts due to the natural yellow hue of folic acid, it may be possible to discern differences in the folic acid-fortified versus non-folic acid-fortified study salts in the participants’ homes. Nevertheless, all clinical specimens will be coded before submission to the laboratories, and the group identity will be concealed from the laboratory technicians and from the data analysis team during the initial stages of data analysis. Participants’ study arms and the identity of the salt types will be revealed only after preliminary analysis of data and following agreement of the co-principal investigators (PI) on the interpretation of study outcomes.

The primary endpoints of the trial are: (1) biweekly disappearance of project-supplied salt from the household; (2) discretionary salt consumption by WRA, as measured by direct observation during in-home weighed food records; (3) changes in markers of folate status (RBC and serum folate, serum homocysteine); (4) changes in markers of iodine intake and status (urinary iodine excretion, serum thyroglobulin); and (5) period prevalence of serious adverse events (SAE). Secondary outcomes include qualitative assessments of salt acceptability, changes in haematological status (haemoglobin (Hb), RBC indices and leucocyte morphology) and possible adverse effects, as assessed from participant reports and by selected metabolic indicators (insulin resistance and metabolomic responses). Possible modifying effects of the intervention that will be explored are: (1) household food insecurity; (2) biomarkers of initial folate, vitamin B_12_ and riboflavin status; and (3) polymorphisms of the genes coding for methylenetetrahydrofolate reductase (MTHFR) and serine hydroxymethyltransferase 1 (SHMT1), which may affect the metabolism of folic acid. We will also assess the effect of free distribution of salt on women’s discretionary salt consumption by monitoring salt intakes and 24-hour urinary sodium excretion before and during the trial.

**Figure 1:**
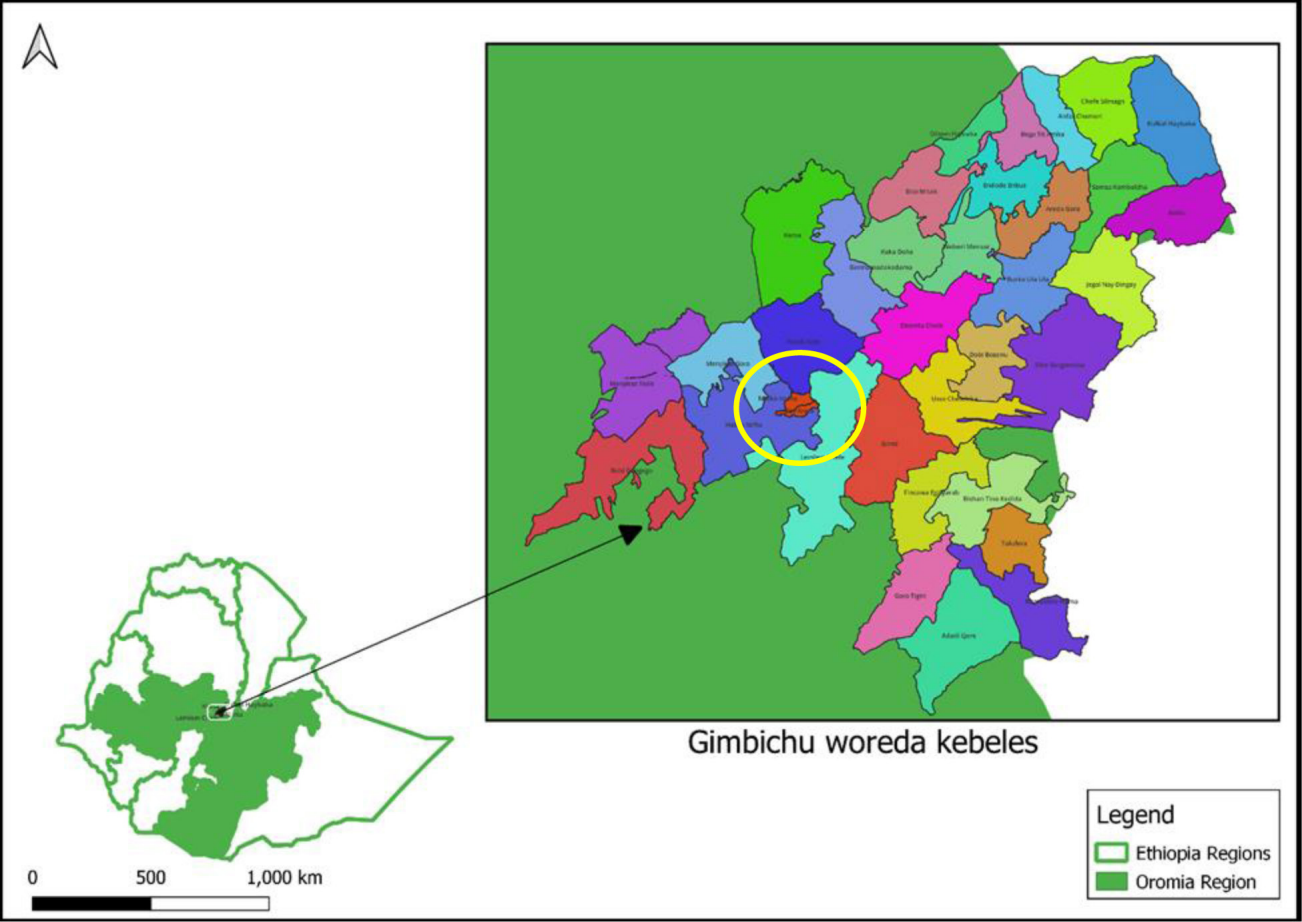
Location of study communities: Gimbichu *woreda*, Oromia Region, Ethiopia.

The study will take place in two rural and two semiurban communities of Gimbichu *woreda*, Oromia Region, Ethiopia (altitude ~2450 m above sea level; figure 1). The total population of the *woreda* is 67 773, of whom ~27% are WRA. According to a preliminary study in the project area, WRA consume a median of 6.6 g discretionary salt per day (95% CI 4.1, 10.2), and, on average, households use approximately 30–47 g salt per day, depending on household size.^25^ A total of 360 eligible women will be randomly assigned to receive one of the three study salts using a block randomisation process.^24^ Based on preliminary studies, salt sharing between households is uncommon, and residents of the study communities are not consuming folic acid-fortified flour (B Tesfaye, unpublished).

The intervention was initiated on 30 April 2024 and data collection should be completed by mid-December 2024.

### Patient and public involvement

As indicated above, the research question was originally articulated by the Ethiopian Ministry of Health. The coauthors of this manuscript designed the study and selected the study communities following consultation with local Ministry of Health authorities. The field staff and local investigators then convened meetings with Ministry of Health personnel and residents of the study communities to explain the rationale, objectives and planned procedures of the study. Residents of the communities were not directly involved in the study design. The plan for dissemination of study results is described below.

### Inclusion, exclusion and withdrawal criteria

Non-pregnant women 18–49 years of age who are using a long-acting contraceptive (injectable or implanted hormonal contraceptive or intrauterine device) and who are not intending to move from the community or become pregnant during the subsequent 6 months will be eligible to participate in the trial if they agree to use only the salt provided by the project for the study duration and provide their informed consent, as indicated by signature or thumb print. In cases where there is more than one woman of reproductive age residing in the household or more than one household residing in the same physical structure, just one woman of reproductive age from that physical location will be randomly selected for participation.

Women who are currently pregnant or at risk of becoming pregnant (ie, not using contraceptives), and those who are unwilling to use the salt provided by the study or are planning to leave the study area within the next 6 months will be excluded from participation. In addition, women will be excluded if they are reportedly suffering from an acute or chronic disease (such as diarrhoea, intestinal malabsorption, chronic febrile illness, or metabolic or cardiovascular disorders) that might affect their dietary intake or folate status, as well as those who are using medications that can interfere with folate metabolism (antacids, inhaled or systemic corticosteroids, hydantoin derivatives, valproic acid derivatives, biguanides, bile acid sequesters, potassium-sparing diuretics). Consistent with national guidelines,^26^ women who have a mid-upper arm circumference <23 cm and are breastfeeding an infant <6 months of age will be referred to the local health system for treatment of undernutrition and will be excluded from the trial. Based on our preliminary studies, we do not expect women to be using vitamin-mineral supplements; but if any women are consuming supplements that contain folic acid, they will be excluded. Women who have hypertension (systolic pressure >140 or diastolic pressure >90) on two occasions on separate days will be referred to the local health centre for further evaluation and treatment and will be excluded from the trial. Women who are found to have macrocytic anaemia (Hb<132 g/L; mean corpuscular volume (MCV)>100 fL) at the time of the initial blood collection will be excluded from the trial and referred to the local health centre for further evaluation and possible treatment with folic acid and/or vitamin B_12_. Women who are found to have microcytic anaemia (Hb<132 g/L; MCV<80 fL) will be referred to the local health centre for presumptive treatment of iron deficiency and will remain eligible for the study. Women with mild, normocytic anaemia (Hb<132 g/L, ≥122 g/L) will be included in the study. Women with moderate/severe normocytic anaemia (Hb<122 g/L) will be excluded from the trial and referred for evaluation. All Hb cut-offs are adjusted for altitude.^27^

Women will be free to withdraw from the study at any time. In addition, women who are absent from the study community and are unable to consume the study-provided salt for more than 28 days (two consecutive or non-consecutive rounds of the biweekly salt delivery) will be dropped from the study. Participants who withdraw or are discontinued after randomisation will not be replaced. All data collected until the time of withdrawal will be included in the intention-to-treat analysis.

### Recruitment of study participants

Using existing household listings, members of the study team will contact a convenience sample of previously identified WRA at their homes to explain the purpose, methods, benefits, inconveniences and risks of the research, and to assess the women’s eligibility and seek their consent to participate. After presenting a disclosure statement and request to proceed with the interview, the data collector will obtain information from verbally consenting women on their age, pregnancy status, contraception practices, education, religion, ethnicity, employment and general health status; household location (GPS coordinates); plans to remain in the study community; housing characteristics (construction materials, water source, sanitary facilities, access to electricity and cellphone service); cellphone number (if available); selected household assets, main source(s) of household income and potential willingness to participate in the subsequent research studies. The data collector will also measure the women’s blood pressure, review the women’s health history and determine their potential eligibility for the study. For women who are potentially eligible to participate, the data collector will describe the purposes of the intervention trial, the study procedures and related risks and benefits, the confidentiality of results and the fact that participation is voluntary. After providing an opportunity for women to consult with family members and ask any questions, the field worker will request signed consent from the women to participate in the intervention trial. The women then will be invited to attend the local health post for additional screening, including a second blood pressure assessment, anaemia assessment and a urinary pregnancy test.

### Informed consent, institutional review board approval and trial registration

For the initial home visit to screen WRA for eligibility, a disclosure statement will describe the components of the interview, and verbal consent will be requested by the data collector before proceeding with the interview. For the intervention trial, signed consent will be required. Printed consent forms prepared in the local language, Afaan Oromo (Oromiffa), will be read aloud by the data collectors to individuals who are considering participation in the study. A participant advocate who is not part of the research team will be present during the consent procedures and will cosign the forms as a witness. The consent form describes how the data will be protected to ensure confidentiality and the possible use of deidentified data and stored biological specimens for future research.

The study protocol was submitted to the institutional review boards (IRB) at the Ethiopian Public Health Institute (EPHI) and the University of California Davis (UC Davis). The EPHI IRB approved the study (including both the preliminary investigations and the intervention trial) on 2 June 2023 (protocol number EPHI-IRB-494-2023). Following its review of the protocol (Project No 2078626-1) and examination of documents from the EPHI IRB, the UC Davis IRB determined on 18 September 2023 that separate approval from UC Davis was not required. The trial protocol has been registered with the ClinicalTrials.gov website (registration number NCT06223854).

Study participants will receive the study salts for free, and they will be compensated in kind with sanitary products, like soaps, and other small (non-monetary) gifts.

### Intervention

Households of WRA will be randomly assigned to one of three study arms to receive salt fortified with: (1) a lower dose of folic acid+iodine, (2) a higher dose of folic acid+iodine or (3) iodine only (comparator group). Based on the results of preliminary studies of discretionary salt intake in the study communities, the amount of folic acid in the two DFS will be 30 ppm for the lower dose arm and 90 ppm for the higher dose arm to provide average additional folic acid intakes of ~200 μg/day or ~600 μg/day, respectively, in the two folic acid groups. These amounts of additional folic acid intake are intended to span a range of intakes that should increase RBC folate concentration, as indicated by a previous folic acid supplementation trial,^28^ and should not result in excessive folic acid intake.^29^ This dose–response design will also permit subsequent modelling to determine the appropriate level of folic acid fortification needed to achieve a desired RBC folate concentration in the local context. We recognise that minor losses of folic acid may occur during storage of DFS-IoFA (B Tesfaye, unpublished),^30^ so the target fortification levels account for this. We also note that folic acid is sensitive to different types of cooking, related cooking temperatures and duration of thermal exposure.^31 32^ Nevertheless, most folic acid is retained during cooking of fortified flours and rice, regardless of the type of cooking, and flour fortification programmes have succeeded in reducing NTDs despite the effects of cooking. Thus, we expect to be able to detect a nutritional response to the folic acid-fortified salt.

**Figure 2:**
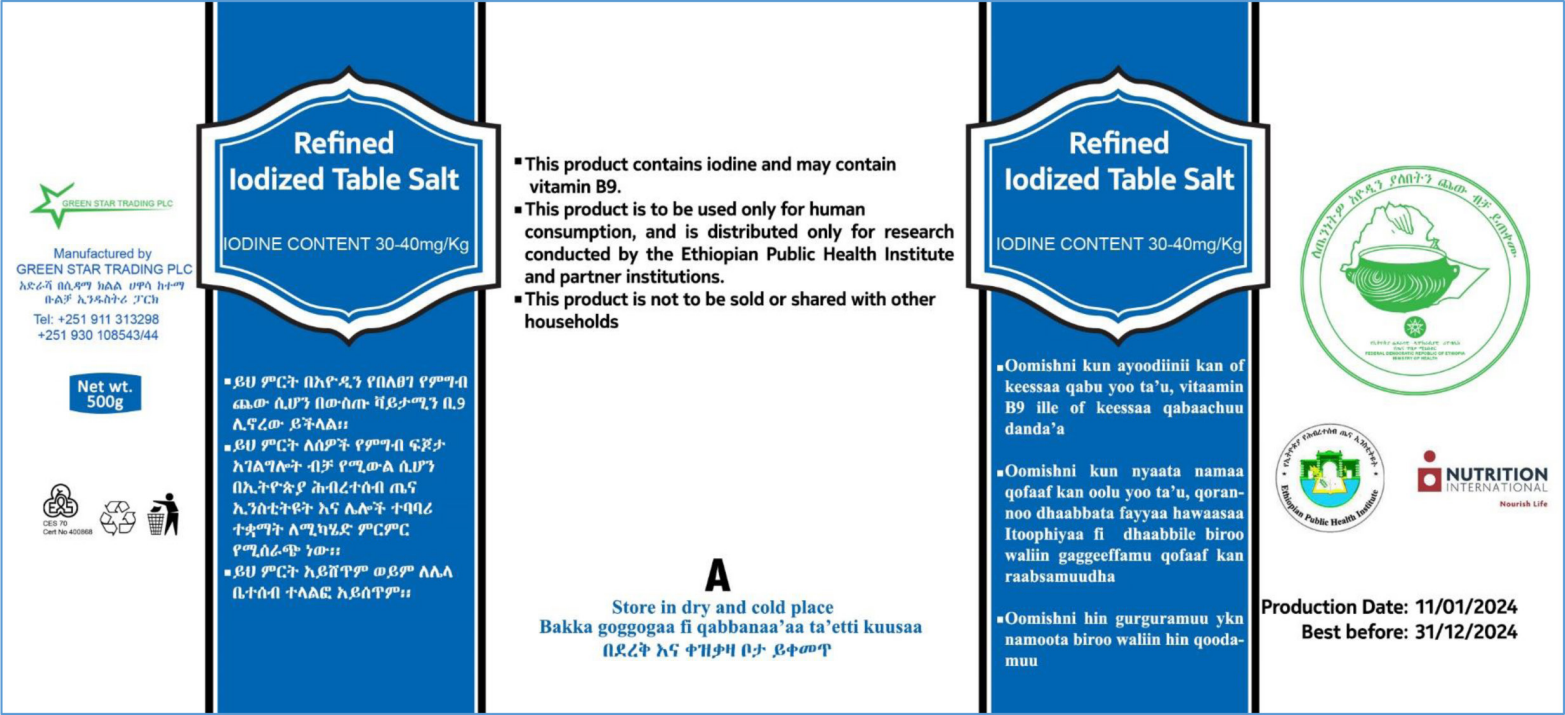
Example of label for salt container.

Following systematic inspection of national salt production facilities, a multistakeholder advisory panel selected Green Star Trading, Hawassa, Ethiopia, to produce the study salts. Project managers from the Nutrition International Ethiopia Country Office will indicate the code letters (A, B or C) to be applied to the three types of salts. These codes will be retained by Nutrition International (NI) and the salt producer, and will be stored in sealed envelopes in locked filing cabinets of the two co-PIs who will not be informed of the salt codes until the data collection and preliminary analyses are completed. The salt production, fortification and quality assurance procedures are described in a separate procedures manual available from NI. As per Ethiopian standards for iodine content, the refined salt used for this study will contain 30–40 ppm iodine as potassium iodate. Both fortificants (iodate and folic acid) will be added simultaneously using the current spray technology used by the production plant. The salt will be packaged in opaque, plastic containers containing 500 g salt, with labels stating that the salt is iodised, may or may not contain vitamin B_9_ and is being distributed for research purchases only (see figure 2 for example of label).

Two staff members of EPHI who are not part of the field study team will receive the salts from the producer and a list of study group assignments from the UC Davis statistician who completes the randomisation list. One of these staff members will place a preprinted adhesive label with the participant ID number and bar code on the container to conceal the letter identifying the type of salt. The second staff member will observe the labelling process and confirm that each participant number is affixed to the correct type of salt. The newly labelled containers of salt will then be stored by participant study number for subsequent distribution.

Project staff members will deliver two containers of the study salts (1 kg salt) to the participating households biweekly for a total of 26 weeks. Any households that use more than the initially delivered amount of salt will be able to request additional salt, as needed. At the time of each home visit for salt delivery, the field workers will request the participants to present their study ID cards to confirm that the correct type of salt is being delivered (by matching the participant identification numbers on the card and the containers of salt). Field staff members will explain repeatedly that: (1) the stored salt should be protected from light and extreme temperatures during storage in the home, and (2) the salt is intended only for consumption by members of the household and should not be shared with other households or used for other purposes.

At the time of each biweekly home visit, the field worker will collect the previously delivered salt containers and any remaining study salt. The field worker will also ask a brief set of questions concerning acceptability of the study salt, use of the salt for special purposes, like preparation of spice mixtures or pickling, use of any other salt (including the amount used and the reason for its use if other salt has been used) and any losses or sharing of the study salt that may have occurred. The leftover salt will be weighed to determine the salt disappearance, which will be expressed both by household and by adult female equivalent based on the theoretical energy requirements of all household members,^33^ considering the household size and composition. During these home visits, the field workers will also check that the women are up to date with regard to their contraception practices and whether they are breastfeeding. Samples of stored salts will be collected at the beginning of the study and every 4 weeks thereafter to analyse the folic acid and iodine concentrations continuously. These results will be monitored over time and will be compared in relation to participant identification number at the end of the trial to confirm correct salt labelling and delivery.

Individual women will participate in the trial for 26 weeks. This period of time has been chosen because it should allow the women to reach a maximum, or near maximum, level of RBC folate concentration following introduction of the intervention, based on the results of a previous supplementation trial.^28^

### Data collection procedures

**Figure 3:**
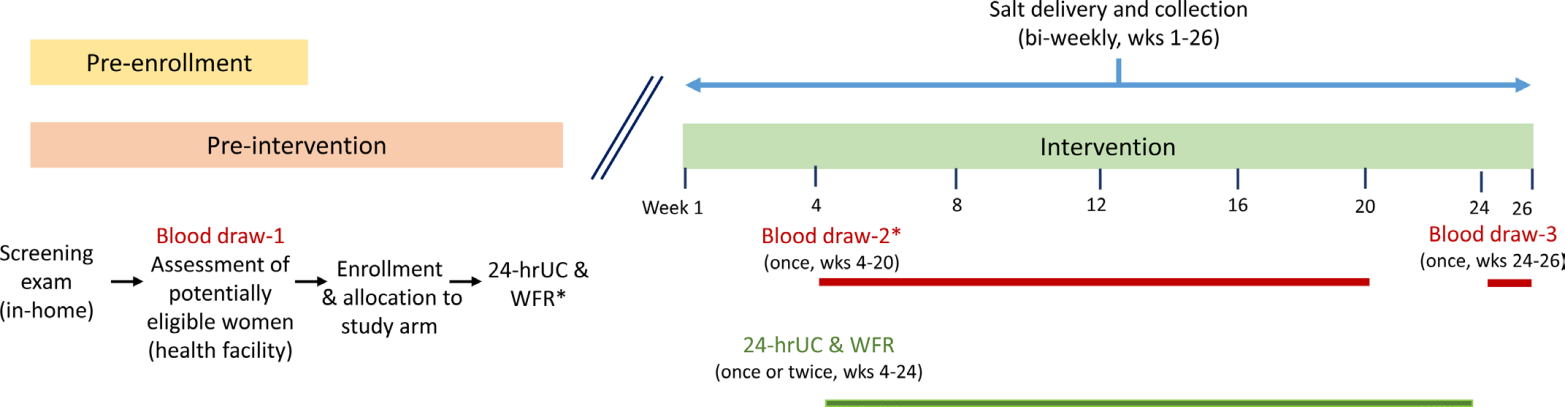
Schedule of data collection activities for individual study participants. *Blood draw 2 will be completed at a randomly assigned time point between weeks 4 and 20 of the intervention period. At the time of each blood draw, anthropometrics and blood pressure will be assessed. 24-hrUC = 24-hour urine collection to assess urinary iodine and sodium excretion; WFR = weighed food record to assess dietary intake.

Within 1 or 2 days of the initial home interviews, potentially eligible, consenting women will be requested to report to one of the local health posts for anthropometric assessment, measurement of blood pressure, fasting blood collection and additional interviews (to determine marital status, birth history, dietary diversity and household food security). The fasting blood specimens (approximately 16 mL whole blood) will be collected for a complete blood count and measurement of RBC folate and serum folate, folate vitamers (including unmetabolised folic acid (UMFA)), homocysteine, vitamin B_12_, methyl malonic acid, thyroglobulin, erythrocyte glutathione reductase activity coefficient, retinol-binding protein (RBP), ferritin, soluble transferrin receptor, C reactive protein, alpha-1-acid glycoprotein, glucose, insulin, genotyping and metabolomics, and to complete a malaria rapid diagnostic test (RDT). Women with a positive malaria RDT will be referred to the local health facility for treatment and will be retained in the study. Blood will be collected again at end line (weeks 24–26 of the intervention) and at a randomly assigned intermediate time point between weeks 4 and 20 of the intervention, using simple random assignment within study arm, such that the blood collections are spread evenly over this interval. (See figure 3 for a summarised schedule of activities.)

All women will participate in dietary assessments—conducted as 24-hour, in-home, weighed food records—to assess discretionary salt intake and will collect 24-hour urine specimens for assessment of urinary iodine and sodium excretion. These studies will be carried out on 1 day before the intervention and 1 day between weeks 4 and 24 of the intervention; a randomly selected subset of 40 women in each study arm will complete these studies a second time during the intervention, 1–2 weeks after the first study, to allow for calculation of intraindividual variation in intakes and urinary excretion.^34^ This is necessary to estimate the distributions of usual discretionary salt intake and urinary sodium and iodine excretion of the study population. Procedures for anthropometry; blood pressure measurement; blood and urine collection, processing and analysis; dietary assessments; and assessments of food security and dietary diversity are described as follows.

#### Anthropometrics

Trained anthropometrists will measure the weight, height, and mid-upper arm, waist and hip circumferences of all women using standard procedures.^35 36^ The results will be expressed as absolute measurements and as body mass index (weight in kilograms/height in metres squared). Weight will be measured in duplicate on a Seca electronic balance (Model 874, Hamburg, Germany) with measurement precision of 0.1 kg. Height will be measured in duplicate using a UNICEF height board (Weigh and Measure; Olney, Maryland, USA), with measurement precision of 0.1 cm. Body circumferences will be measured using flexible, non-stretchable measuring tapes, with measurement precision of 0.1 cm.

#### Blood pressure measurement

Blood pressure will be assessed at the time of the screening interview in the women’s homes and at each of their early morning clinic visits for blood drawing. In particular, following a rest period of at least 15 min while the women are seated, blood pressure will be measured twice at least 3 min apart using an OMRON silver automated blood pressure monitor (OMRON model BP5250). All procedures will adhere to the recommendations of the American Heart Association Council on High Blood Pressure Research.^37^ High blood pressure will be defined as systolic pressure >140 or diastolic pressure >90. If a woman is found to have hypertension during each of the baseline assessments, she will be referred to the local health centre for evaluation and treatment, and she will be excluded from the study.

#### Blood sample collection and processing

**Figure 4:**
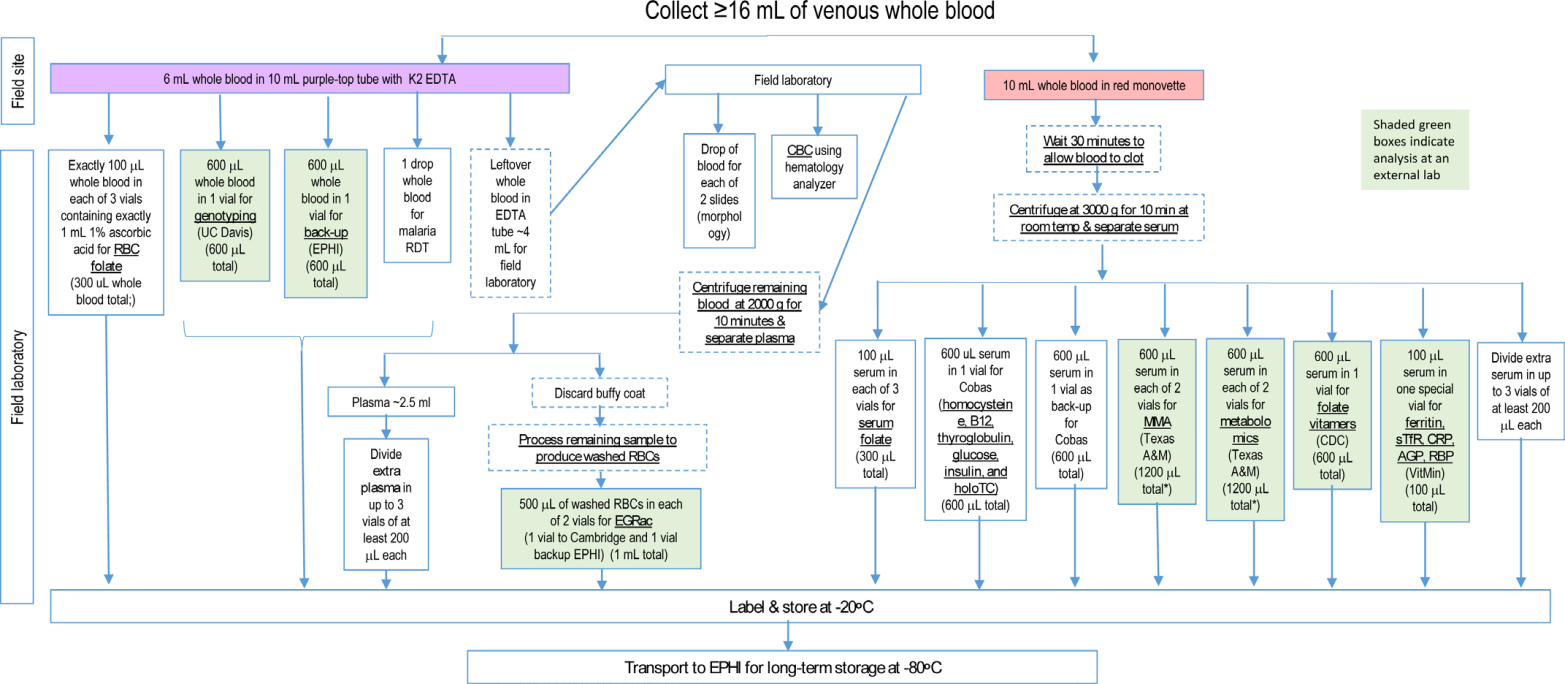
Blood collection and processing flow chart. *Second vial for methylmalonic acid (MMA) and metabolomics will be stored at the Ethiopian Public Health Institute (EPHI) in case of loss in transit (two vials of 600 μL to be sent to Texas A&M for MMA and metabolomics). AGP, alpha-1-acid glycoprotein; CBC, complete blood count; CDC, Centers for Disease Control and Prevention; CRP, C reactive protein; EGRac, erythrocyte glutathione reductase activation coefficient; holoTC, holotranscobalamin; RBC, red blood cell; RBP, retinol-binding protein; RDT, rapid diagnostic test; sTfR, soluble transferrin receptor.

On the days of blood collection, eligible women will be requested to arrive at the local health post after fasting for at least 8 hours. Approximately 16 mL blood will be collected aseptically from an antecubital vein by experienced phlebotomists using vacutainer blood collection systems. Collection tubes will be protected from light and processed within 30 min of collection. The resulting specimens will be organised for different laboratory analyses, as illustrated in figure 4. Specifically, 6 mL blood will be collected into a potassium EDTA tube for the malaria diagnostic test; haemolysates for RBC folate determination; and whole blood for a complete blood count, blood smear for morphological examination and preparation of washed RBCs for determination of the erythrocyte glutathione reductase activation coefficient (to assess riboflavin status). The remaining blood will be collected into serum separator tubes and allowed to clot for 30 min (in a cooler and shielded from light). The blood specimens for serum samples will be spun at 3000 g for 10 min at the field site using a portable centrifuge, and the serum will be transferred into multiple cryotubes, as shown in figure 4. All collection and storage tubes will be carefully labelled with preprinted labels containing the participant’s identification number, collection date, type of specimen and a bar code to simplify future identification. One 50 µL serum specimen will be kept in a special, prelabelled tube for shipment to the VitMin Laboratory in Germany, as described below. The storage tubes for folate analyses will be wrapped in aluminium foil to protect them from light exposure, and all tubes except the ones for RBC folate determinations will be transferred immediately to a −20°C freezer at the field site. The samples will be transported to the EPHI laboratory in Addis Ababa in a portable freezer (−20°C) within 1 week. The samples for RBC folate will be processed in the field before freezing, as described below. Once the sample vials arrive at the EPHI laboratory, they will be stored in a freezer at −80°C until laboratory analysis. Each type of specimen (ie, for a particular analysis) will be grouped together in separate storage boxes for easy identification at the time of analysis or shipment to an external laboratory. Samples will be stored until the end of the trial so that all samples from each woman can be analysed together during the same analytical run.

#### Twenty-four-hour urine collection, processing and storage

24-hour urine specimens will be initiated during the days of the dietary observations in the participants’ homes using the procedures described for the US National Health and Nutrition Examination Survey.^38^ If the participant is menstruating, the urine collection will be rescheduled. Shortly after the dietitian arrives at the home, the participant will be asked to urinate and discard the urine specimen. The time of that void will be recorded, and all subsequent urine excreted during the next 24 hours will be collected in a plastic bottle by the participant and stored in a cold box provided by the study team. On the following day, the dietitian will return to the participant’s home and request the participant to urinate at the same time that the collection was initiated the previous day. This last specimen will be retained in the collection bottle. The dietitian will then record the time of the final urination, measure the total volume of urine and prepare separate aliquots for transfer to the EPHI laboratory. Samples will be considered acceptable for analysis if the total urine volume is >400 mL, the woman is not menstruating, the collection duration is ≥22 and ≤26 hours and there is no reported loss of any urine specimen during that interval.

#### Dietary intake assessments

Trained data collectors will measure all food and salt intakes during full-day assessments using weighed dietary records collected by direct observation of food preparation and consumption in the participants’ homes. For these studies, the data collectors will arrive at the participants’ homes early in the morning, in time to observe preparation of the first meal of the day. For all meals prepared or consumed during the day, all foods and condiments included in mixed preparations will be weighed to 0.1 g precision before they are added to the cooking pot. The final weight of the completed preparation will be recorded before and after cooking. The amount of each preparation served to the woman and any leftovers will be weighed to calculate the amounts consumed, which will then be expressed as a per cent of the total postcooking weight and multiplied by each raw ingredient weight to determine the amounts of each ingredient that were consumed. Discrete food items, such as pieces of fruit or biscuits, will be weighed separately before serving; any leftovers will be weighed, as described above, to determine the amounts consumed. The data collectors will return to the homes the following day to inquire whether any foods were consumed during the previous night after the dietitian left the home. Any foods consumed during the night (after the last evening meal of the previous day and before the current day’s breakfast meal) will be recorded by recall history using local utensils and the diet balance to estimate the portion sizes. During the same visit, the data collectors will complete the urine collections, as described above.

The total daily consumption of individual foods will be summed and subsequently converted to folate (and other nutrient) intakes using the Ethiopian food composition table (FCT), supplemented with information from the Kenyan, West African, South African, Indian and US Department of Agriculture (USDA) FCTs, as needed. Discretionary salt intake will be measured as all salt (including salt in spice mixtures) added to mixed dishes during preparation or added at the time of consumption. Salt transfer to the preparations or plates will be recorded by weighing the salt storage vessel before and after transfer using a balance with 0.1 g precision.

#### Assessment of household food insecurity and women’s dietary diversity

Household food insecurity will be assessed using the Household Food Insecurity Access Scale published by the US Agency for International Development Food and Nutrition Technical Assistance project.^39^ Household food insecurity will be considered a potential modifying factor for response to folic acid fortification.

The women’s dietary diversity (DD-W) will be assessed using the open recall and recording form suggested by the Food and Agriculture Organization for the 10 food group indicators.^40^ DD-W will be assessed for the previous day at the time of each dietary assessment and at every other salt delivery contact (ie, every 4 weeks). The number of food groups consumed will be averaged for each woman. Minimum dietary diversity will be defined as consumption of at least five food groups, and consumption of leafy green vegetable and legume food groups will be assessed separately and related to folate status.

#### Laboratory analyses

Pregnancy test. A pregnancy test will be completed using a urine dipstick at the time of the screening examination at the local health facility using test materials to be procured through the Ethiopian Ministry of Health.

Malaria RDT. The presence of malaria antigens (*Plasmodium falciparum* and *P. vivax*) in whole blood will be assessed using a drop of blood from the venous blood collection and a point-of-care malaria RDT. The Ethiopian Ministry of Health currently uses the Standard Diagnostics immunochromatographic assay (SD Rapid Test, www.standardia.com).

Haematology assessments. Haematology assessments will comprise a complete blood count and RBC indices, to be completed at the field site using an automated haematology analyser (Sysmex Model XQ-320; Sysmex Europe, Norderstedt, Germany), and a blood film coloured with Wright stain for subsequent microscopic examination, focusing primarily on the presence of hypersegmented polymorphonuclear leucocytes.

RBC folate. RBC folate concentration will be measured at EPHI using a microbiological assay,^41^ as adapted by the US CDC.^42^ For this assay, exactly 100 µL of whole blood collected in EDTA will be diluted in 1 mL 1% ascorbic acid solution (1% weight:volume) to haemolyse the RBCs and maintain folate in a reduced state. The haemolysate will then be promptly frozen and stored at −20°C at the field site until transported to a −80°C freezer at EPHI within 1 week. The microbiological assay of the haemolysate will be performed in quadruplicate using a chloramphenicol-resistant strain of *Lactobacillus rhamnosus* and a 5-methyl-tetrahydrofolate calibrator. Quality control pools for low and high whole blood folate concentration will be analysed with each assay. For each analytical run, a standard growth curve of the organisms will be completed. The growth of the organisms after incubation at 37°C for 42–45 hours will be read as turbidity at 590 nm wavelength using a microplate reader (BioTek Elx808; Agilent, Santa Clara, California, USA). The haematocrit determined on the same blood specimen will be used to calculate the RBC folate concentration.

Serum folate. Serum folate will be measured at EPHI using the same general procedures as described for RBC folate. However, the serum aliquot will be frozen immediately after centrifugation of the blood specimen and will be diluted only after the stored aliquot is thawed for analysis.

Serum homocysteine. Homocysteine will be measured at EPHI on a COBAS 6000 analyser using an enzymatic cycling assay (Roche, reference number 05385415190) that assesses the co-substrate conversion product.

Serum vitamin B_12_ and transcobalamin. Serum vitamin B_12_ and holotranscobalamin will be measured at EPHI on a COBAS 6000 analyser using competitive binding assays (Roche, reference numbers 07212771 and 08717028190).

Serum glucose and insulin. Serum glucose and insulin will be measured at EPHI on a COBAS 6000 analyser using a hexokinase enzymatic method for glucose (Roche, reference number 04404483190) and an electrochemiluminescence immune assay for insulin (Roche, reference number 12017547122). The homeostasis model assessment of insulin resistance (HOMA-IR) will be calculated as described by Matthews *et al*^43^ using the standard ratio formula: HOMA-IR=(FI×FG)/22.5, where FI is fasting serum insulin (pmol/L) and FG is fasting serum glucose (mmol/L).

Serum thyroglobulin. Serum thyroglobulin will be measured at EPHI on a COBAS 6000 analyser using an immunoassay (Roche, reference number 08906556190).

Serum methylmalonic acid (MMA). MMA in serum will be measured at the Texas A&M University Institute for Advancing Health Through Agriculture using the gas chromatography/mass spectrometry (GC/MS) protocol recommended by CDC.^44^ For this assay, MMA is extracted from serum along with an internal standard (d3MMA) using a commercially available strong anion exchange resin. The extracted MMA is derivatised with cyclohexanol to form a dicyclohexyl ester. The derivatised samples are injected onto a gas chromatograph, and the effluents are monitored with a mass detector using selected ion monitoring. Results are quantified by internal calibration using peak area ratios of MMA and the internal standard.

Serum UMFA. Serum UMFA and other folate vitamers will be measured at the Division of Laboratory Sciences, National Center for Environmental Health, US CDC, using isotope dilution liquid chromatography-tandem mass spectrometry according to the method of Fazili *et al*.^45^

Serum RBP, ferritin, soluble transferrin receptor, C reactive protein and alpha-1-acid glycoprotein will be analysed at the VitMin Laboratory in Willstaett, Germany. These specimens will be stored in special tubes provided by the VitMin Laboratory. The samples will be analysed by multiplexed immunoassay, as described previously.^46^ RBP will be expressed in relation to retinol using a regression equation developed during preliminary studies (M Woldeyohannes, unpublished).

Metabolomics. Untargeted metabolomics will be completed at Texas A&M University. Serum specimens will be analysed by GC/MS following extraction with methanol and internal standards, followed by derivatisation using methoxyamine hydrochloride and N-methyl-N-trimethylsilyl-trifluoroacetamide, as described by Trezzi *et al*.^47^ Metabolite detection and identification will be performed using MetaboliteDetector software.^48^

Genetic polymorphisms. Analysis of polymorphisms of the genes coding for MTHFR and SHMT1 will be completed at the UC Davis Real-time PCR Research and Diagnostics Core Facility using the methods described previously.^49^ Briefly, DNA will be extracted from whole blood using a QIAcube HT automated nucleic acid work station (Qiagen, Valencia, California) according to the manufacturer’s instructions for the QIAamp 96 DNA QIAcube HT Kit (Qiagen). Quantitative PCR (qPCR) will be completed using the rs1801133 single nucleotide polymorphism (SNP) for MTHFR and the rs1979277 SNP for SHMT1, both of which are available from Thermo Fisher Scientific. The Thermo Fisher Design and Analysis software identifies the genotype based on programmed positives selected before the qPCR analysis is initiated.

Urinary sodium, potassium and iodine. Urinary sodium and potassium concentration will be measured at EPHI on a COBAS 6000 analyser using ion selective electrodes (Roche, reference number ISE NA-U ACN 29071). Urinary iodine concentration will be measured at EPHI using the Sandell-Kolthoff reaction.^50^

#### Laboratory quality assurance

The EPHI Food and Nutrition Laboratory will be responsible for the folate microbiological assay and the analyses of urinary iodine. For each of these analyses, the laboratory prepares pooled reference materials with low, medium and high concentrations. These pools are analysed 30 times and compared with appropriate external standards. The same pooled samples are analysed with each subsequent daily analytical run, and the results must fall with the range (±2 SD) of the original analyses for the day’s results to be considered acceptable. The laboratory will participate in the CDC Performance Verification Program for the Folate Microbiologic Assay. Under this external quality assurance (EQA) programme, CDC provides 10 samples per quarter in duplicate, and the participating laboratory returns results to CDC after each of the four challenges. CDC returns a brief summary report after each challenge and a comprehensive annual performance report showing the measurement imprecision and the per cent difference compared with the CDC Clinical Laboratory Improvement Amendments (CLIA)-approved folate microbiological assay. The laboratory also participates in the CDC EQA programme for urinary iodine procedures (Ensuring the Quality of Urinary Iodine Procedures).^51^

The EPHI Clinical Laboratory will complete analyses of serum vitamin B_12_, holotranscobalamin, homocysteine, thyroglobulin, glucose, and insulin and urinary sodium and potassium, all of which are completed on the COBAS 6000 analyser using special reagent kits and controls for each analyte. Each day, the COBAS analyser is standardised for each analyte to ensure conformity with acceptable result ranges, as specified by the manufacturer. This laboratory participates in the Oneworld Accuracy EQA programme.^52^ Under this programme, the laboratory analyses five unknown samples for each analyte every 4 months, and the results are compared for multiple consensus laboratories. The EPHI laboratory has been fully accredited for analyses of vitamin B_12_, glucose, and insulin and urinary sodium and potassium.

#### Assessment of SAEs

SAEs are defined as any illness or disorder that results in death or long-term disability, or requires an overnight stay in a clinical facility, regardless of the nature of the condition.^53^ All SAEs will be recorded during the biweekly home visits and reported within 48 hours to the co-PIs, who will classify them as being ‘likely to be related to the intervention’ or ‘unlikely to be related to the intervention’. The co-PIs will share these findings with the IRB on a monthly schedule.

We have established an external Data and Safety Monitoring Board (DSMB) to review whether any SAEs occur more frequently in any of the study groups. The DSMB comprises an Ethiopian physician, a statistician, a nutritionist, a staff member of the Ethiopian Food and Drug Authority and a person with expertise in research ethics. A list of all SAEs will be recorded continuously for periodic review by the DSMB statistician, who is not participating in the core study team. The DSMB statistician will aggregate the SAEs by study arm for review by the DSMB. The review frequency will depend on the number of new SAEs occurring over time. The DSMB will have the authority to interrupt or stop the trial if there is a significant excess number of SAEs that may be related to the intervention in either folic acid group.

### Sample size estimates

The sample size estimation is based on the number of women required to detect a clinically significant change in their RBC folate concentration in response to the intervention. According to the 2016 Ethiopian National Micronutrient Survey,^13^ the estimated mean RBC folate concentration among WRA is 507 nmol/L. If we assume a coefficient of variation of 35%, as has been observed in most other studies (as summarised in table 1),^2854^,^56^ the corresponding SD for Ethiopian women would be 177.5 nmol/L. To detect an effect size of 0.5 SD units (ie, 89 nmol/L), which would represent the difference in the change in mean RBC folate concentration from baseline to 6 months between women in the control group and women in the group receiving salt fortified with 200 μg folic acid per day, 85 participants per group are necessary. A difference of 89 nmol/L is equivalent to 18% of the expected mean RBC folate concentration at baseline, which is more conservative than the 27% change observed among women receiving a 100 µg folic acid supplement in a study by Hao *et al*.^28^ This calculation assumes an alpha of 0.05 and power of 0.90. After adjusting the sample size to account for the three-cell design, and an additional 15% to account for possible attrition, the total required sample size per group is 120 participants (ie, 360 participants total). This sample size will allow for comparisons between any two of the study arms.

**Table 1:** Published data regarding population RBC folate concentration and responses to intervention trials

Study	Folic acid dose	n	Mean (or geometric mean)	SD (or CI)	Estimated SD	Coefficient of variation, CV (%)	% change from baseline	Ref
Ethiopia national survey	–	1121	511	(487, 527)	340	67.1	–	13
Guatemala national survey	–	1448	727	(711, 738)	262	36.0	–	54
Hao baseline	–	339	594	(572, 618)	216	36.3		28
Hao 6 months	100 μg/day	339	760	(731, 789)	272	35.6	27.9	28
Hao 6 months	400 μg/day	338	1036	(997, 1076)	370	35.8	74.4	28
Johansson baseline	166 μg/day		577	93		16.1	–	55
Johansson 3 months	166 μg/day		694	154		22.2	20.3	55
Johansson baseline	355 μg/day		784	238		30.3	–	55
Johansson 3 months	355 μg/day		987	167		16.9	25.9	55
Lamers baseline	–	34	668	(593, 752)	236	35.4	–	56
Lamers 24 weeks	400 μg/day	34	1290	(1200, 1370)	496	38.4	93.1	56

RBCred blood cell

Based on information on urinary iodine concentration among WRA published in the 2016 National Micronutrient Survey (median=96.7 µg/L, IQR 57.6–170.5),^13^ this sample size will permit detection of a 35 µg/L difference in urinary iodine concentration in either of the arms receiving folic acid-fortified salt compared with the control group (alpha=0.05; beta=0.90). Because we will obtain information on mean 24-hour urinary iodine concentration and total daily urinary iodine excretion rather than just single urine samples, we should be able to detect an even smaller effect size than would be possible with just a single sample.

### Data management and statistical analysis

For most types of data, information will be collected using automated (tablet-based) data entry with range checks and consistency checks incorporated into the data entry software. One exception is the dietary intake data, which will be recorded first on paper forms for preliminary review before entering the consolidated food intake data into the database.

Open Data Kit (ODK) software will be used for primary data collection.^57^ The records will be submitted to a web-accessible ODK aggregate database installed in a dedicated, secure server at EPHI. All sets of up-to-date raw data will be archived in comma-separated value (CSV) format. The archived data will be the aggregated data from each record that formed the basis for editing and analysis. First, all identifiable information will be removed and individual records will be coded just by the participant identification numbers. Deidentified raw data will be retained at EPHI and transferred to UC Davis weekly for editing, generating additional data reports and archiving.

The data managers at EPHI and UC Davis will jointly prepare the data editing syntax, which will be accessible to the appropriate co-investigators, along with the raw data and any data correction forms. Raw CSV data will be exported to STATA V.17 software, and data review and editing will be performed using the same software, for which STATA syntax will be developed. For errors, mistakes or missing data identified during data review, a separate data correction form will be completed and signed by the data collector, supervisor and field coordinator.

A detailed statistical analysis plan will be completed and posted publicly before the analysis is initiated. All study personnel will remain blinded to intervention group assignment (A, B or C) until investigators have reached agreement on result interpretation. Briefly, the data analysis will consist of descriptive analyses of sociodemographic data, anthropometry, blood pressure and vitamin and mineral status at baseline, followed by group-wise comparisons of change in folate and iodine status using both intention-to-treat and per protocol analyses. All data collected from women who subsequently withdraw from the study will be retained and included in the intention-to-treat analyses. Group-wise comparisons of possible adverse metabolic effects will be completed initially just in the control group versus higher folic acid group.

Variable distributions will be described using means, prevalences and percentiles, as appropriate. Generally, intervention effects will be assessed using analysis of covariance models to estimate group mean differences for continuous outcomes controlling for baseline measurement. Models will be repeated in minimally adjusted and multivariable models to increase the precision of intervention effect estimation. A similar approach will be taken for categorical outcomes using log-binomial or modified Poisson regressions (dependent on model convergence) to estimate prevalence ratios across groups. Prespecified effect modification (eg, by baseline RBC folate concentration, markers of vitamin B_12_ status, genetic polymorphisms and selected household socioeconomic and demographic variables) will be assessed by incorporating interaction terms into the models, followed by regions of significance or stratified analyses to understand the nature of effect modification.

In addition to measuring the impact of the intervention, we will model the levels of folic acid intake necessary to achieve selected RBC folate concentrations. This will be done by using the distribution of RBC folate in the control group to characterise what distribution shift would be necessary to move nearly all (>95%) of the women above the target concentration to minimise the risk of NTDs. Next, the differences in final distributions between the control group and the two intervention groups will be used to interpolate what folic acid fortification level would achieve the desired distribution shift.

### Management of trial

Major decisions on scientific aspects of the trial will be the responsibility of the co-PIs and co-investigators, who will also recruit, train and supervise the other members of the study team. Day-to-day operations will be managed jointly by the field coordinator from EPHI and a postdoctoral research fellow from UC Davis. The field team will be organised into separate domains of activity, including participant screening, enrolment and anthropometry; blood collection and processing; distribution of salt and assessment of compliance; dietary assessment and 24-hour urine collections; blood collection and preliminary processing; laboratory analysis; data analysis; and administration and logistics. Study participants will be recruited over a period of 3 months, and all primary data and specimen collections should be completed within 9 months.

Senior members of the EPHI field team will be involved in different aspects of institutional and community sensitisation, as appropriate. Ministry of Health and other public sector administrators have been contacted at the regional, zonal, woreda and kebele levels to explain the research, address any concerns and facilitate project-related activities. The health facility heads in the study area have authorised data collection activities and access to local health facilities. Separate meetings are being convened with individual (political, religious and traditional) community leaders and potential study participants and their families.

All data collection and laboratory procedures will conform to written instructions compiled in a standard operating procedures manual. A separate statistical analysis plan will be prepared before initiation of the data analysis. Data entry forms will be drafted, field tested and revised accordingly. The forms will be prepared in English, translated into the local language (Oromiffa) and back-translated to English. In most cases, these forms will then be converted to a digital data entry platform, as described above.

Quality assurance procedures related to the salt production and stability are described above. The field supervisors, field coordinator and postdoctoral fellow will supervise all day-to-day data collection procedures at the field site. In the case of the salt distribution, the field coordinator will visit all study homes on at least two occasions throughout the trial, according to a predetermined schedule. The purposes of the visits will be to confirm the date of the last field worker visit and check the participants’ identification cards and labels on the salt delivered, and to review the household salt storage and use practices.

The project has two advisory committees, each with specific roles and unique composition. In particular, a Scientific Advisory Group comprising global technical experts meets periodically to review the research design and study progress. Individual members are also available, as needed, to address specific questions. A second advisory group, the Project Advisory Committee (PAC), is composed mostly of representatives of national Ethiopian agencies that will help guide the implementation of the project and facilitate coordination among the different national agencies. Members of the PAC are representatives of the following government agencies: the Nutrition Coordination Office of the Ministry of Health; the Food, Beverage, and Pharmaceutical Industry Development Institute of the Ministry of Industry; the Ministry of Trade; the Ethiopian Food and Drug Authority; the Ethiopian Standards Authority; and a member of the Ethiopian House of People’s Representatives. Through these agencies, the project will align with the Ethiopian Food and Nutrition Strategy, which is overseen by the National Food and Nutrition Program Management Steering Committee. EPHI is responsible for providing technical support and serving as chair for research, monitoring and evaluation of the national Food and Nutrition Strategy.

### Safety considerations

Concerns have been raised in the scientific literature regarding several possible adverse effects of excessive folic acid intake, and these issues need to be considered by folic acid fortification programmes. One possible concern relates to the potential ‘masking’ of the haematological effects of vitamin B_12_ deficiency by folic acid administration, thereby leading to delayed recognition of vitamin B_12_ deficiency and its related neurological consequences. Indeed, the US Institute of Medicine (IOM) established its safe upper intake level (UL) for folate based on ‘suggestive evidence that folate intake [5 mg/d] may precipitate or exacerbate neuropathy in vitamin B12-deficient individuals’.^58^ The IOM UL was set at 1 mg/day based on an uncertainty factor applied to the lowest observed adverse effects level of 5 mg/day. This uncertainty factor provides a considerable margin of safety. The European Food Safety Authority used the same information and conceptual framework,^59^ and established the same UL as the IOM. However, as later summarised by Berry,^60^ several authors have disputed these analyses, because there does not, in fact, appear to be any difference in the rate of neurological disease progression due to vitamin B_12_ deficiency in relation to the level of folic acid intake. These authors concluded that there is no justification for withholding folic acid because of the possible risk of vitamin B_12_ deficiency. Instead, vitamin B_12_ status should be assessed and managed independent of folate intake. We note that the most recent nationally representative nutrition survey in Ethiopia found a 9% prevalence of vitamin B_12_ deficiency (defined as serum vitamin B_12_<191 pg/mL) among WRA.^61^

Other potential adverse effects of folic acid that have been discussed most commonly include a possible increased cancer risk due to suppressed immune function and impaired insulin sensitivity. Other diseases, like allergy and cognitive disorders, have also been postulated, although there is very little information available on these latter issues. With regard to cancer, it is well accepted that low folate intake is an independent risk factor for several types of cancer.^62^ On the other hand, some animal studies and secondary analyses of human trials have suggested that high folic acid intakes may increase the risk of colorectal cancer.^63^ However, these latter findings are inconsistent, and most trials and retrospective studies have not identified an increased cancer risk.^63^ The results of studies of the effect of folic acid on immune function are also inconsistent, and the doses of folic acid that were assessed were considerably greater than what might be delivered by a fortification programme.

With regard to folate status and glucose metabolism, an observational study in India found that 6-year-old children of women who had both low vitamin B_12_ status and high folate status during pregnancy had lower insulin sensitivity, as measured by the HOMA-IR. The authors suggested that the combination of low maternal vitamin B_12_ status and high folate status may contribute to type 2 diabetes in India.^64^ Other investigators completed a maternal folic acid intervention trial among pregnant Nepali women and applied a similar HOMA-IR method to assess insulin sensitivity in children aged 6–8 years who were born to those women. This group of researchers likewise found greater insulin resistance among the children of vitamin B_12_-deficient mothers, but there was no relationship between either maternal folate status or folic acid supplementation during pregnancy and the children’s HOMA-IR results.^65^

As reviewed in a recent paper on the safety of folic acid fortification,^66^ several expert groups have examined the foregoing concerns regarding possible adverse effects of folic acid supplementation and fortification. These expert groups include the National Toxicology Program of the US National Institute of Environmental Health Sciences,^67^ a National Institutes of Health/FDA/USDA workshop on the metabolic and clinical effects of excess folates/folic acid,^63^ the UK Scientific Advisory Council on Nutrition,^68^ the Norwegian Scientific Committee for Food Safety^69^ and New Zealand’s Office of the Prime Minister’s Chief Science Advisor.^70^ These groups all report that there are no consistent adverse effects of folic acid, and in the few cases where such events have occurred the dose of folic acid that was consumed was considerably greater than what might be provided by a fortification programme. Given the confirmed benefits of folic acid fortification for reducing the risk of NTDs, the unanimous consensus of these groups is that the benefits of folic acid fortification outweigh any unconfirmed potential risks of adverse effects and that folic acid fortification programmes should proceed, while appropriate authorities continue to monitor for any possible adverse effects.

Based on the results of our preliminary studies of 100 women’s discretionary salt intakes in the study communities, we estimate that women in the higher folic acid fortification arm whose usual salt intakes are at the 95th percentile (ie, 10.2 g salt per day) will consume 918 µg folic acid per day from salt.^25^ Thus, the vast majority of women in the study population will consume less than the UL of 1000 µg/day; and we conclude that the proposed level of folic acid fortification should not produce any adverse effects. Nevertheless, we will monitor continuously for any adverse events, and we will assess the women’s insulin sensitivity and metabolic responses to the intervention.

## Dissemination of results

We plan to publish the results of the study in leading open access nutrition and/or public health scientific journals, applying authorship guidelines published by the International Committee of Medical Journal Editors^71^; and we will present the results at major nutrition conferences globally and nationally. EPHI will also disseminate the study results through the project’s PAC and via scientific briefs targeted to national authorities and key stakeholders.

Final databases with deidentified personal information will be archived in the Ethiopian National Data Management Center at EPHI and will be made available to scientists from other institutions once the primary manuscripts have been submitted for publication. Individuals who request access to the data set or stored biological specimens will be required to describe the objectives of their analyses, the variables and/or specimens requested and any plans for collaboration with the core research team.

Study participants will be informed of abnormal clinical (blood pressure, anthropometric) and laboratory (anaemia, vitamin and mineral status, hyperglycaemia) results and will be referred to the local health facilities for further evaluation and treatment, as appropriate.
